# *Plasmodium* APC3 mediates chromosome condensation and cytokinesis during atypical mitosis in male gametogenesis

**DOI:** 10.1038/s41598-018-23871-9

**Published:** 2018-04-04

**Authors:** Richard J. Wall, David J. P. Ferguson, Aline Freville, Blandine Franke-Fayard, Declan Brady, Mohammad Zeeshan, Andrew R. Bottrill, Sally Wheatley, Andrew M. Fry, Chris J. Janse, Hiroyuki Yamano, Anthony A. Holder, David S. Guttery, Rita Tewari

**Affiliations:** 1 School of Life Sciences, Queens Medical Centre, University of Nottingham, Nottingham, UK; 2 Nuffield Department of Clinical Laboratory Science, University of Oxford, John Radcliffe Hospital, Oxford, UK; 3 Leiden Malaria Research Group, Parasitology, Center of Infectious Diseases, Leiden University Medical Center (LUMC), Leiden, The Netherlands; 4 Protein and Nucleic Acid Chemistry Laboratory, Centre for Core Biotechnology Services, University of Leicester, Leicester, UK; 5 Department of Molecular and Cell Biology, University of Leicester, Leicester, UK; 6 UCL Cancer Institute, University College London, London, UK; 7 The Francis Crick Institute, London, UK; 8 The Leicester Cancer Research Centre, College of Life Sciences, University of Leicester, Leicester, UK; 9 Present Address: The Wellcome Trust Centre for Anti-Infectives Research, School of Life Sciences, University of Dundee, Dundee, UK

## Abstract

The anaphase promoting complex/cyclosome (APC/C) is a highly conserved multi-subunit E3 ubiquitin ligase that controls mitotic division in eukaryotic cells by tagging cell cycle regulators for proteolysis. APC3 is a key component that contributes to APC/C function. *Plasmodium*, the causative agent of malaria, undergoes atypical mitotic division during its life cycle. Only a small subset of APC/C components has been identified in *Plasmodium* and their involvement in atypical cell division is not well understood. Here, using reverse genetics we examined the localisation and function of APC3 in *Plasmodium berghei*. APC3 was observed as a single focus that co-localised with the centriolar plaque during asexual cell division in schizonts, whereas it appeared as multiple foci in male gametocytes. Functional studies using gene disruption and conditional knockdown revealed essential roles of APC3 during these mitotic stages with loss resulting in a lack of chromosome condensation, abnormal cytokinesis and absence of microgamete formation. Overall, our data suggest that *Plasmodium* utilises unique cell cycle machinery to coordinate various processes during endomitosis, and this warrants further investigation in future studies.

## Introduction

Cell division in the malarial parasite, *Plasmodium* spp., involves distinct events not seen in most other eukaryotes. Despite a detailed morphological description of the cell division process and the subcellular structures involved^1^, the molecular mechanisms and key components required for the different stages are not well understood. It has also been found that a number of highly conserved cell cycle regulators, such as cell division cycle 25 (CDC25), CDC14 and classical cyclins are absent from *Plasmodium*^2–4^.

Mammalian cell division involves an open mitosis, where mitotic spindle formation is accompanied by nuclear envelope disintegration and subsequent partition of the cytoplasm during cytokinesis^5^. In contrast, *Plasmodium* undergoes a closed mitosis, which proceeds with karyokinesis (nuclear division) without concomitant cytokinesis^6,7^. In many organisms the cell cycle is spatially and temporally coordinated with the centrosome duplication cycle. During mitosis, the centrosome serves as a microtubule organising center (MTOC), with the separated pair of duplicated centrosomes forming the two poles of the mitotic spindle^8^. However, *Plasmodium* has structurally distinct centrosomes that lack classical centrioles. Instead, spindle microtubules originate from an MTOC referred to as a centriolar plaque (CP) that resembles the spindle pole body (SPB) found in yeasts and *Dictyostelium*^9^. In *Plasmodium*, the CP functions as a major site for microtubule formation, determining microtubule movement during mitosis^6^.

During the *Plasmodium* life cycle, there are two atypical mitotic processes: one that resembles endomitosis occurs during asexual multiplication, for example, during blood stage schizogony^6,7^, and another that occurs during the sexual stage - the formation of microgametes (male progenitor sex cells) in the mosquito midgut^10^. During schizogony, genome duplication and segregation proceed via the formation of an intra-nuclear spindle without disintegration of the nuclear membrane^1,7^ resulting in a multinucleated syncytium called a schizont. In microgametogenesis, exposure of the male gametocyte to mosquito midgut factors results in ‘activation’ of the microgametocyte, which undergoes three rounds of rapid genome duplication from haploid to octaploid, followed by simultaneous chromatin condensation and nuclear budding. Each condensed haploid nucleus and associated MTOC, together with a basal body, axoneme and flagellum, is incorporated into the microgamete, which egresses from the main cellular body in a process termed exflagellation^10^. Eight motile microgametes are released 8 to 12 minutes post activation (mpa)^10^. Exflagellation in *Plasmodium* is tightly regulated by protein phosphorylation, with key roles for protein kinases, including calcium dependent protein kinase 4 (CDPK4), mitogen-activated protein kinase 2 (MAP2) and SR protein kinase (SRPK), as well as metallo-dependent protein phosphatase 1 (PPM1)^2,11^.

One essential component that drives the cell cycle, and particularly mitosis, in many eukaryotic systems is the Anaphase Promoting Complex/Cyclosome (APC/C). The APC/C is a multi-subunit E3 ubiquitin ligase that promotes cell-cycle progression by covalently tagging regulators such as securin and cyclin B1 with ubiquitin leading to their proteolysis by the proteasome^12,13^. The mammalian APC/C has 14 core components, and several key adaptor subunits, including cell division cycle protein 20 (CDC20) and the related CDH1^13,14^. Intriguingly, only four APC/C components have been identified as coded by the *Plasmodium* genome: APC10, APC11 and APC3 (a tetratricopeptide repeat [TPR] containing subunit), along with CDC20^15^.

In *Plasmodium*, there is a single orthologue of CDC20, which is the only member of the APC/C components functionally characterised to date^16^. *Plasmodium* CDC20 is dispensable during asexual multiplication in blood stage schizogony but has an essential role in microgametogenesis^16^. In other eukaryotes, APC10 (also known as Destruction of cyclin B protein 1 or Doc1) promotes substrate binding and increases the processivity of substrate ubiquitylation^17,18^; APC11 is a RING-H2 zinc finger protein that forms a tight complex with APC2 and constitutes the catalytic core of the APC/C; and APC3 (also known as CDC27) forms a homo-dimer with one molecule binding, via its TPR repeats, to a C-terminal Ile-Arg (IR) motif of CDC20/CDH1 and the other binding to the C-terminal IR motif of APC10, which together with CDC20/CDH1 forms a co-receptor for the D-box of APC/C substrates^14^. APC3 also interacts with mitotic checkpoint complex (MCC), through CDC20, suggesting that it may play a role in controlling checkpoint function^19,20^. As APC3, APC10, APC11 and CDC20 are key subunits involved in APC/C-dependent substrate recognition and ubiquitylation in eukaryotes, we hypothesised that these *Plasmodium* homologues might form a ‘minimal’ complex for ubiquitylation and/or control the *Plasmodium* life cycle.

Here, we investigate the localisation and function of APC3 in schizogony and male gametogenesis using gene disruption and conditional knockdown in the rodent malaria parasite *Plasmodium berghei*. We demonstrate that APC3 is associated with the MTOC-like centriolar plaque during schizogony and male gametogenesis, but surprisingly is not associated with the other APC/C components. However, it is required for chromosome condensation and cytokinesis, but not DNA replication, during microgamete formation.

## Results

### *Plasmodium* APC3, APC10, APC11 and CDC20 do not form detectable complexes

**Figure 1:**
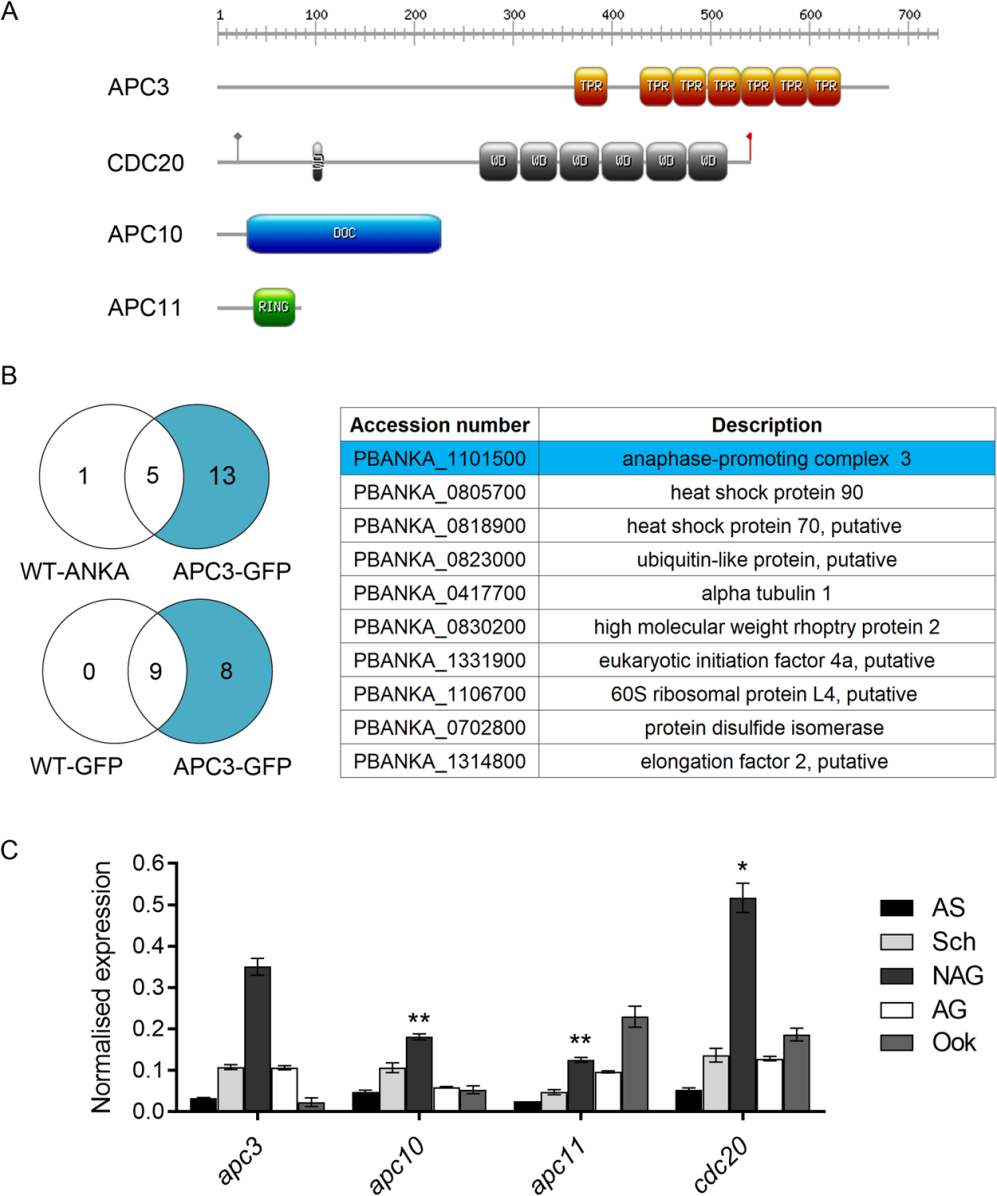
Protein domain analysis, complex formation and transcription. (**A**) Protein domains of the four putative APC/C components. TPR: Tetratricopeptide repeat, WD: WD40 repeat, RING: RING finger. Grey circle: KEN box, Red flag: IR motif. (**B**) Analysis of APC3-GFP by mass spectrometry following immunoprecipitation from a parasite lysate using gametocytes activated for 7 min. Venn diagrams display mean totals of shared and unique proteins compared with WT-GFP (constitutively expressing GFP) and WT-ANKA (without GFP) lines. Table lists unique proteins found only in APC3-GFP samples. Full results can be found in Supplementary Table S1. For APC3-GFP parasite generation see Supplementary Fig. S1. (**C**) Normalised transcript levels of *apc3, apc10, apc11* and *cdc20* throughout the wild type parasite life cycle analysed by qRT-PCR. Data were normalised against two endogenous control genes, *arginine-tRNA synthetase* and *hsp70*. Each bar is the mean of three biological replicates ± SEM. All asexual blood stages: AS; schizonts: Sch; non-activated gametocytes: NAG; activated gametocytes: AG; ookinete: Ook. Unpaired *t*-tests were performed between *apc3* and the other APC/C components in NAG. *p < 0.05, **p < 0.001. See also Supplementary Figs. S1 and S2; Supplementary Tables S1–3.

Only a small number of APC/C components have been identified in the apicomplexan parasite *Plasmodium*, namely: APC3, APC10, APC11 and CDC20^15^. We began by examining their putative structures and testing whether they form a complex in *Plasmodium*. Homology modelling suggested significant similarity with known APC/C component structures^21–23^ (Supplementary Fig. S1a). However, primary sequence analysis indicated that *Plasmodium* APC3 is highly divergent from known orthologues^15^, despite retaining the seven predicted conserved TPRs that bind to the IR motif (Fig. 1A). Since the IR motif is absent from *Plasmodium* APC10 this raises the question of whether and how APC10 might interact with APC3 (Fig. 1A). To investigate whether the proteins form a complex in *Plasmodium*, immunoprecipitation was performed from transgenic parasite lines expressing C-terminal GFP-tagged versions of the endogenous APC3 and CDC20 sequences (Fig. 1B and Supplementary Fig. S1b)^16^. Successful integration of the targeting vector downstream of *apc3* was shown by integration PCR and Pulse Field Gel Electrophoresis (PFGE), while the presence of the APC3-GFP fusion protein (111 kDa) was confirmed by Western blot of gametocyte cell lysate (Supplementary Fig. S1c–e). Immunoprecipitation was performed using activated gametocytes since both *Plasmodium* APC3 and CDC20 are expressed highly in male gametocytes at this stage^16^. During gametogenesis, anaphase-related processes are predicted to start around 7 to 8 minutes post-activation (mpa) when nuclear replication has occurred and chromosomes start to condense, making this the optimum point to investigate APC/C complex formation. Mass spectrometry analyses of immunoprecipitates revealed no co-precipitation of other APC/C components with either APC3-GFP or CDC20-GFP (Fig. 1B and Supplementary Fig. S1f,g; Supplementary Tables S1 and S2). Quantitative RT-PCR (qRT-PCR) analysis, as well as previous RNA-seq data^24^, suggest that these components have subtly different expression patterns during the parasite life cycle (Fig. 1C). We also attempted to examine protein-protein interactions among *Plasmodium* APC/C components identified to date using a yeast two-hybrid assay (Supplementary Fig. S2a–c). Surprisingly, APC10 self-interaction, presumably dimerization, was observed, but no other interactions between the four *Plasmodium* APC/C components were detected (Supplementary Fig. S2a–c), although expression of APC3 was very poor. All together, these results strongly suggest that, unlike in other eukaryotic cells, *Plasmodium* APC/C components may act independently during the cell cycle.

### *Plasmodium* APC3 associates with centriolar plaques resembling SPBs in schizonts

**Figure 2:**
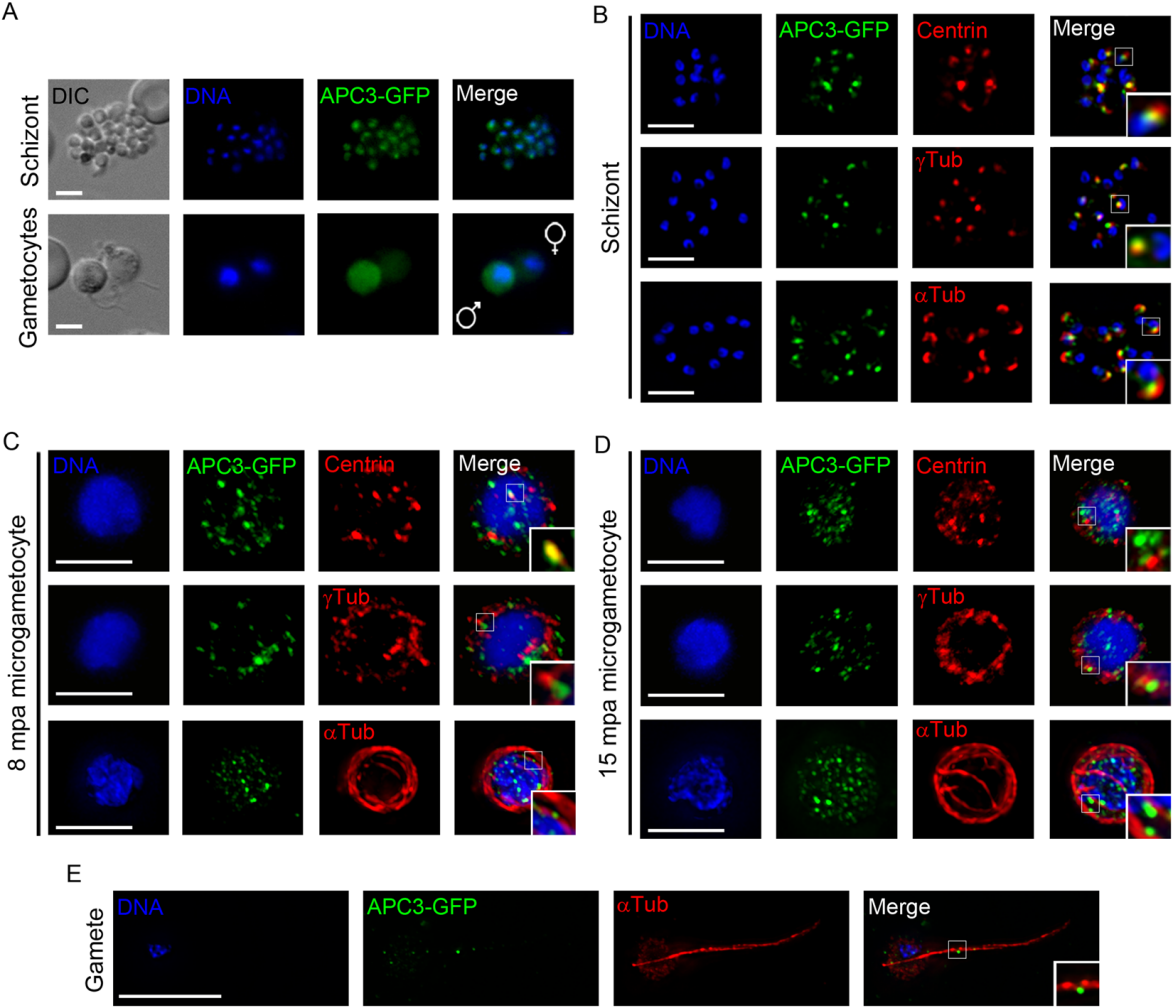
Localisation and expression of APC3-GFP in mitotic cells. (**A**) Live imaging or (**B** to **E**) fixed IFA of APC3-GFP with centrin, α-tubulin (α-Tub) or γ-tubulin (γ-Tub) antibodies in schizonts, microgametocytes (gametocytes) and gametes. DIC: Differential interference contrast, Merge: GFP and Hoechst. Scale bar = 5 µm. For APC3-GFP parasite generation see Supplementary Fig. S1.

APC3 is a conserved component of the centrosome that controls the metaphase-anaphase transition during mitosis^25^. However, since *Plasmodium* has CPs instead of a centriole-containing centrosome, we explored the localisation of the APC3 protein in dividing cells using the APC3-GFP transgenic parasite line (Supplementary Fig. S1b–e). Live cell imaging revealed cytoplasmic localisation of APC3-GFP in schizonts and microgametocytes (Fig. 2A) but no expression was observed at any other stage of the life cycle examined. Using immunofluorescence microscopy and deconvolution analysis in both asexual and sexual dividing stages (schizonts and male gametocytes), APC3-GFP was found to localise to specific foci in the cytoplasm. Co-staining with antibodies against the known centrosomal markers, centrin and ɣ-tubulin, revealed that in schizonts a single focus of APC3-GFP was present associated with each nucleus, which partially co-localised with a single focus of centrin and γ-tubulin; this is highly persuasive of an association of APC3 with the CPs in this stage of the life cycle (Fig. 2B).

During male gametogenesis, multiple MTOC-like structures were observed with centrin and γ-tubulin antibodies. A few of these exhibited partial colocalisation with APC3-GFP foci but the majority did not (Fig. 2C and D). These features suggest that *Plasmodium* differs from models such as yeast and mammal cells where only a single MTOC is observed in interphase. Since the process of cell division is very dynamic and fast during male gametogenesis it is possible that APC3 may not associate strongly with these multiple MTOCs. Staining of parasites with antibodies to α-tubulin revealed the microtubule-based flagellar axoneme coiled around the nucleus in microgametocytes at 8 mpa^26^, with very little colocalisation with APC3-GFP. However, when axonemes were extended in the flagellated, mature microgametes, more than 50% of APC3-GFP could be observed as a single focus midway along the axoneme (Fig. 2E; Supplementary Table S3).

### All *Plasmodium* APC/C proteins (APC3, APC10 and APC11) are likely essential for schizogony, whilst APC3 has an additional essential function during male gamete formation

To elucidate the function of the three *Plasmodium* APC/C components, we attempted to delete each gene (*apc3*, *apc10* and *apc11*) on 4 separate occasions each by replacement with a *Toxoplasma gondii dhfr/ts* selectable marker using a double homologous recombination strategy in asexual blood stage parasites (Supplementary Fig. S3a,b). In contrast to our previous study showing that CDC20 was not essential for blood stage schizogony^16^, we were not able to delete any of these other genes suggesting that all three proteins are likely essential during blood stage schizogony.

Since APC3 TPR motifs recruit the substrate-binding coactivators CDC20 and CDH1^27^, and APC3 is a key centre for cell cycle regulation, we used a promoter swap strategy to knock-down *apc3* expression and study its function in male gametocytes and during microgametogenesis (Supplementary Fig. S4a).

**Figure 3:**
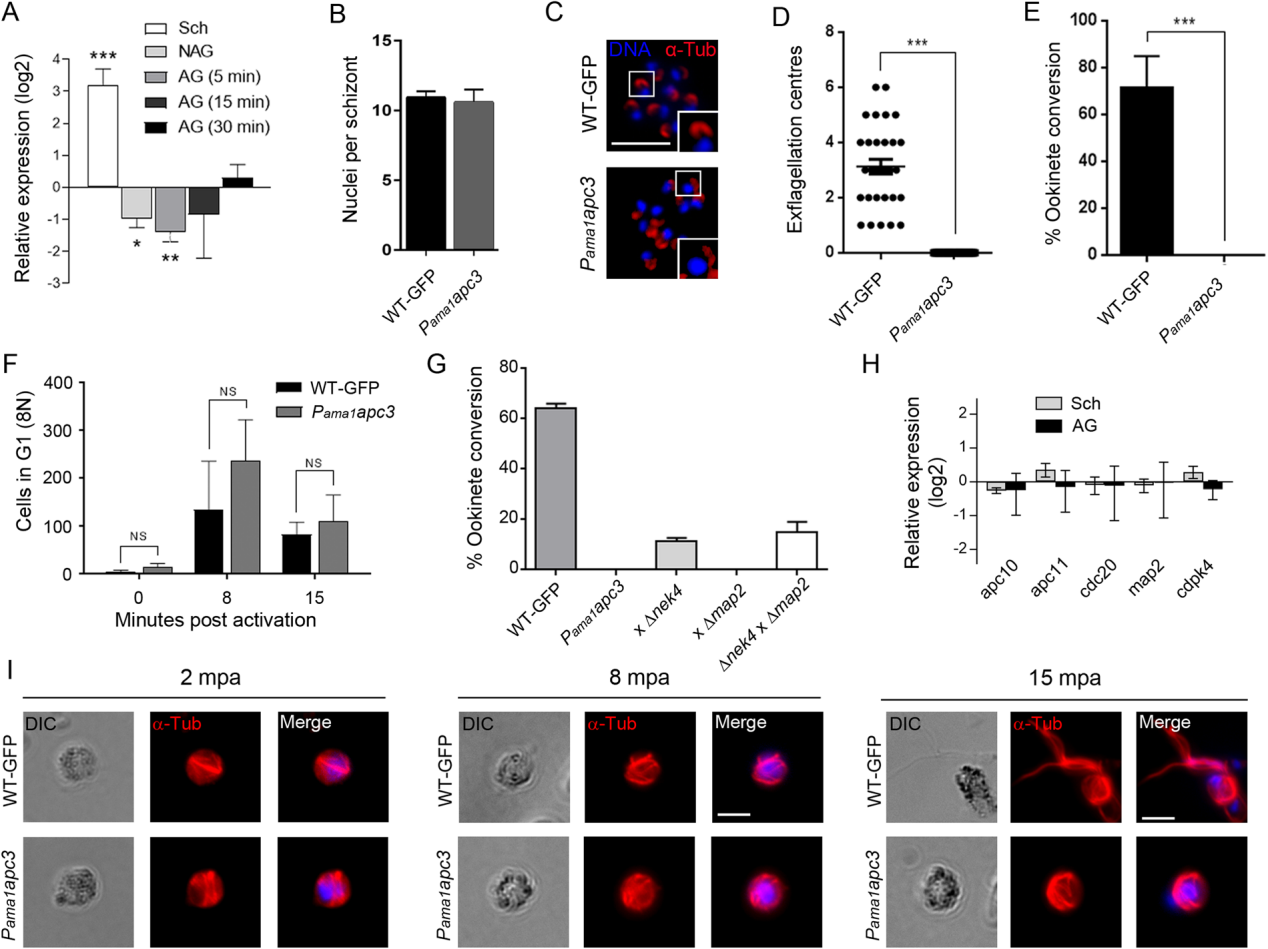
Conditional knockdown of *apc3* during gametogenesis. (**A**) Relative *apc3* transcript levels normalised against two endogenous control genes, *arginine-tRNA synthetase* and *hsp70*, in *P*_*ama1*_*apc3*. n = 3 biological replicates ± SEM. Schizonts: Sch; non-activated gametocytes: NAG; activated gametocytes: AG. Unpaired t-test was performed: * p < 0.05, ** p < 0.01, *** p < 0.001. (**B**) Number of nuclei per schizont. n = 3 independent experiments (30 schizonts per experiment) + /− SEM. (**C**) IFA of α-tubulin in schizonts. Scale bar = 5 µm. (**D**) Number of exflagellation centres per field at 15 mpa. n = 3 independent experiments (10 fields per experiment) + /− SEM. Unpaired t-test was performed: *** p < 0.001. (**E**) Ookinete conversion from zygotes. n = 6 independent experiments (>50 cells per experiment). Unpaired t-test was performed: *** p < 0.001. (**F**) FACS analysis of cells in G1 phase of the cell cycle for 0, 8 and 15 mpa. n = two independent experiments + /− SEM. Unpaired t-test was performed. (**G**) Genetic complementation of *P*_*ama1*_*apc3*. Combinations of WT-GFP*, P*_*ama1*_*apc3* or *P*_*ama1*_*apc3* with either male (∆*map2*) or female (*∆nek4*) mutants were activated in ookinete medium. n = > 100 cells from 3 independent experiments. (**H**) Normalised relative transcript levels of selected genes related to APC/C and cell cycle regulation during microgametogenesis by qRT-PCR. n = mean from 3 replicates + /− SEM. (**I**) IFA of α-tubulin in *P*_*ama1*_*apc3* or WT-GFP microgametocytes activated for 2, 8 or 15 min. Differential intereference contrast (DIC) and merged image with 4′,6-diamidino-2-phenylindole (DAPI) DNA staining are also shown. Scale bar = 5 µm. For *P*_*ama1*_*apc3* parasite generation see Fig. S4.

For this purpose, the endogenous *apc3* was placed under the control of the *ama1* promoter since *ama1* is expressed in schizonts but has low expression during gametogenesis^28^. Two independently derived clones, known collectively as *P*_*ama1*_*apc3*, with the *ama1* promoter integrated upstream of the *apc3* coding sequence were generated, as confirmed using integration PCR, Southern blot and PFGE (Supplementary Fig. S4b–d). In these parasites *apc3* gene expression was maintained in the asexual blood stages but reduced in gametocytes, as confirmed by qRT-PCR (Fig. 3A). Asexual parasite development was normal and the localisation of centrosomal markers, centrin and γ-tubulin, was not affected in *P*_*ama1*_*apc3* schizonts (Fig. 3B,C and Supplementary Fig. S4e). In contrast, in the *P*_*ama1*_*apc3* parasite line, *in vitro* activation of microgametocytes for up to 30 min did not result in microgametogenesis as microgamete formation and exflagellation were not observed (Fig. 3D). Further development including *in vitro* fertilisation and zygote/ookinete formation did not occur and no oocysts were formed when infected blood was fed to *Anopheles* mosquitoes *in vivo* (Fig. 3E and Supplementary Fig. S4f). These data indicate that APC3 has an essential role during mitosis in both blood stage schizogony and microgametogenesis, in contrast to CDC20 that has an essential function only in male gametogenesis^16^. Further analysis of genome replication in activated *P*_*ama1*_*apc3* gametocytes showed that the DNA content progressed to octoploidy (8 N) after 8 mpa, similar to the control, indicating successful DNA replication had occurred (Fig. 3F and Supplementary Fig. S4g,h). We next investigated whether the observed phenotype in gamete formation was a male-specific defect by performing a genetic cross of *P*_*ama1*_*apc3* with a parasite that produces fertile micro- and infertile macro-gametes (∆*nek4*). The partial rescue to form zygotes/ookinetes is consistent with the *P*_*ama1*_*apc3* defect being in microgametes alone (Fig. 3G). Crosses of parasites with a fertile macro- and infertile micro-gamete did not rescue the phenotype, confirming that *P*_*ama1*_*apc3* parasites are defective along the male line.

To determine if *apc3* knockdown affected other APC components and known mitotic kinases like *cdpk4* and *map2* involved in gametogenesis, we examined their transcriptional profile in *P*_*ama1*_*apc3* parasites. This analysis showed that transcript levels of the other APC/C components and mitotic kinases were unaffected in activated *P*_*ama1*_*apc3* gametocytes (Fig. 3H). Staining of centrosomal markers, as well as α-tubulin, was also unaffected in *P*_*ama1*_*apc3* microgametocytes (Supplementary Fig. S4i–k). This suggests that while APC3 is partially localised to the CPs during mitosis, formation of MTOCs in schizonts and gametocytes is not APC3-dependent. Staining of the flagellum with an α-tubulin antibody at multiple time points showed that, while control wildtype GFP-expressing (WT-GFP) male gametes had a fully formed flagellum and had egressed after 15 mpa, the microgametes had not egressed from the gametocyte body of *P*_*ama1*_*apc3* parasites despite axoneme formation (Fig. 3I). This phenotype is similar to that of ∆*cdc20* and ∆*map2* parasites which exhibit complete ablation of exflagellation, and confirms an essential role for APC3 in microgamete formation^16^.

### *Plasmodium* APC3 is required for chromosome condensation and cytokinesis

**Figure 4:**
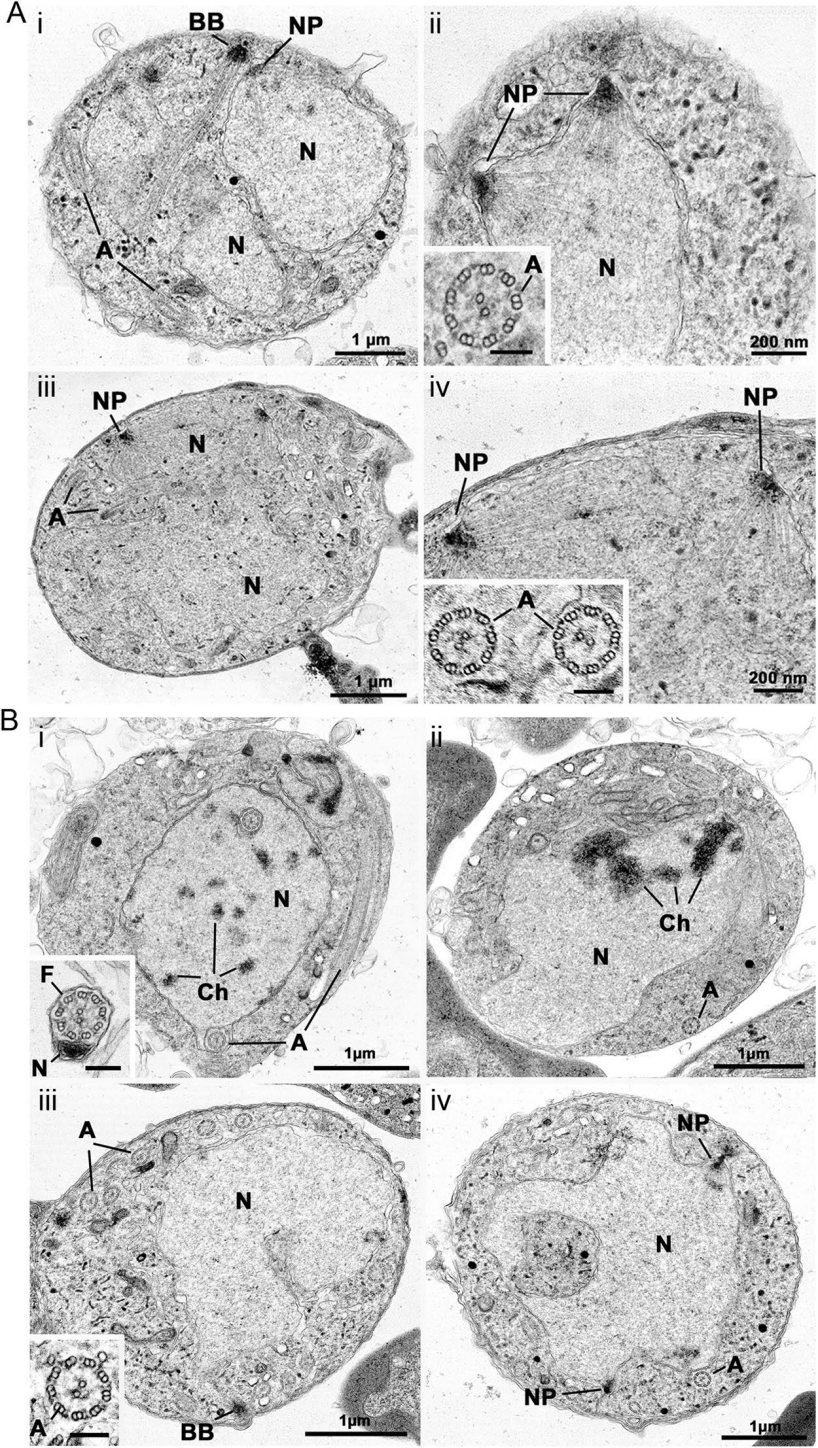
Electron microscopy of microgametocytes. (**A**) WT-GFP (i, ii) and *P*_*ama1*_*apc3* (iii, iv) microgametocytes at 8 mpa. (**B**) WT-GFP (i, ii) and *P*_*ama1*_*apc3* (iii, iv) microgametocytes at 15 mpa (i, iii) and 30 mins (ii, iv). A: axoneme, BB: basal body, Ch: condensed chromatin/chromosome, F: flagellum, N: nucleus, NP: nuclear pole. Bar on inserts = 100 nm. See also Supplementary Fig. S4.

To gain deeper insight into the morphological changes in the *P*_*ama1*_*apc3* mutant, we examined the activated *P*_*ama1*_*apc3* microgametocytes using transmission electron microscopy at 8 mpa, 15 mpa and 30 mpa time points. The ultrastructure of wild type and mutant parasites revealed that at 8 mpa microgametocytes in the *P*_*ama1*_*apc3* mutant appeared similar to WT-GFP parasites with a single, large nucleus containing multiple nuclear poles and radiating microtubules, while the cytoplasm contained basal bodies and axonemes with the 9 + 2 microtubular structure (Fig. 4A, Table 1). However, at 15 mpa, while a substantial number of control WT-GFP microgametocytes showed evidence of chromatin condensation within the nucleus and formation of free microgametes, there was no evidence of chromatin condensation or microgamete formation in *P*_*ama1*_*apc3* microgametocytes (Fig. 4B, see insert, Table 1). These morphological defects were similar to those observed in ∆*cdc20* parasites where we showed a blockage of nuclear division at the nuclear spindle/kinetochore stage^16^. Since *Plasmodium* retains its nuclear envelope during closed mitosis and chromosomes do not condense until late in the cell cycle, these observations indicate that disruption of either *apc3* or *cdc20* blocks late stages of cell cycle progression; whereas DNA replication is not affected. These ultrastructural changes are also similar to those of mutant parasites lacking MAP2, but distinct from those in parasites lacking CDPK4, which show defects in both DNA replication and exflagellation^16,29^.

**Table 1:** Quantitation of the ultrastructural features of wild type and mutant parasites based on stage of microgametocyte development.

Sample	Nuclear features			9 + 2 axonemes	Free microgametes^4^
	No specific^1^	Early/mid^2^	Late^3^		
WT 8 min	56	44	0	+	−
*Pama1apc3* 8 min	62	36	0	+	−
WT 15 min	39	39	22	+	++
*Pama1apc3* 15 min	55	45	0	+	−
WT 30 min	35	41	24	+	+++
*Pama1apc3* 30 min	59	41	0	+	−

Nuclear features observed by electron microscopy at 8, 15 and 30 mpa based on the examination of 100 microgametocytes identified by axonemes at each time point. The stage identified were nuclei with:

^1^No specific features in the plane of section.

^2^Early/mid stage with nuclei with nuclear poles and spindle microtubules with attach kinetochores.

^3^Late stage with the nucleus exhibiting areas of condensed chromatin.

^4^No free microgametes were observed in the mutant at any time point while increased numbers were observed between 15 and 30 mpa in the wild type.

## Discussion

*Plasmodium*, which belongs to the group *Apicomplexa*, undergoes an unusual set of mitotic division processes, has an atypical repertoire of cell cycle regulators, and possesses morphologically different MTOC-like structures compared to most other eukaryotes^2,4,6,9,11,16^. These observations call into question how conserved are their mechanisms of cell cycle control. Here, we have addressed this question through analysis of the *Plasmodium* APC/C components, and APC3 in particular. Our data reveal different gene expression profiles for the four *Plasmodium* APC/C components, a lack of typical APC/C binding domains, and an inability to detect interaction of APC3 with other APC/C components by co-immunoprecipitation pull down or yeast two-hybrid assays. Together, these data suggest that APC3 operates in *Plasmodium* in a manner distinct from that in the well-described pathways in other eukaryotes. These atypical functions may also be present in certain other parasites and plant species. Indeed, in some cases these functions are not required at all. Consistent with this idea, other closely related apicomplexan parasites appear to no longer require a conventional APC/C, with *T. gondii* lacking APC3^15^, and *Babesia*, *Theileria* and *Giardia* lacking all known APC/C subunits^15,30^.

During microgametogenesis, we observed multiple MTOC-like structures by staining with antibodies against centrin and γ-tubulin. The requirement for multiple MTOCs may reflect the rapid DNA replication and segregation during this stage when the genome is replicated three times within 8 to10 min. The different forms of MTOC, including centrosomes, apical complexes and centriolar plaques, are a distinct feature of Apicomplexan parasites such as *Plasmodium*^9^. Novel bipartite centrosomal components^31^ and an essential factor for chromosome replication (ECR1)^32^ have been reported in *Toxoplasma*. None of the APC components have been studied in any other Apicomplexan parasite. Whilst in eukaryotic cells, both APC3 and CDC20 are localised to the centrosome^25,33–35^, here we found that APC3 localised to multiple foci in microgametes, but showed little obvious co-localization with the MTOCs. The function and composition of these APC3 foci remain to be determined.

Here, we show that all three *Plasmodium* APC/C components (APC3, APC10, and APC11) are likely to be essential for asexual multiplication during blood stage schizogony. This is in contrast to CDC20, which is not essential at this stage. Functional studies using conditional gene knockdown also revealed an essential role for APC3 in gametogenesis during chromosome condensation and cytokinesis. This observation closely resembles the requirement for CDC20, as well as one of the atypical MAP kinases (MAP2)^16^. Interestingly, APC/C activity has an important role in MTOC assembly in *Schizosaccharomyces pombe*, with MTOC formation blocked in cells with mutated APC/C components^33^. In *Arabidopsis thaliana*, which undergoes open mitosis^36^, APC3 deletion led to severe perturbation of gametogenesis and mitotic progression^37^. The *A. thaliana* APC/C regulates expression of CYCB1;1 and DUO POLLEN1 (DUO1), two proteins required for cell division in male gametophytes^38^. Although *Plasmodium* undergoes closed mitosis, deletion of APC3 shows similar defects during gametogenesis and cytokinesis. Furthermore, the lack of chromosome condensation supports the conclusion that cells reach G2 phase but do not progress to anaphase. This shows similarity to another protozoan parasite, *Trypanosoma brucei*, in which loss of APC3 leads to parasites with two kinetoplasts and an enlarged single nucleus consistent with cells reaching G2 but not anaphase^39^. Knockdown of the other *T. brucei* APC/C components revealed no such phenotype, suggesting they are not required for APC3 function or cell cycle progression^39^.

The detailed mechanisms of cell cycle control in *Plasmodium* may well be different from those in model organisms, for example the presence of only a small subset of APC/C components^15^ is already indicative of an atypical APC/C. In addition, a number of other key cell cycle regulators are also missing in *Plasmodium*, such as the protein phosphatases CDC25 and CDC14, and classical cyclins^3,4,16^. However, it remains possible that not all APC/C components have been identified in *Plasmodium* due to a lack of sequence or structural similarity with known components in other organisms. Indeed, although phylogenetic analysis showed that CDC25 is missing from *Plasmodium*, several cyclin dependent kinases (CDKs) are present that have residues in the catalytic site which are normally subjected to phosphorylation and dephosphorylation, and so may be targeted by a structurally-distinct set of enzymes in this organism^3^.

The *Plasmodium* genome encodes an expanded family of plant-like calcium dependent protein kinases (CDPKs), of which CDPK4 has been shown to be essential for male gametogenesis, affecting DNA replication and exflagellation. A recent study showed that CDPK4 coordinates various cell cycle events during male gametogenesis in *Plasmodium*^40^. Here, nuclear content analysis revealed that activated *P*_*ama1*_*apc3* male gametocytes reached octoploid (8 N) DNA content but cytokinesis leading to haploid (1 N) gametes did not occur. In contrast, a ∆*cdpk4* mutant did not undergo nuclear replication. This difference suggests that APC3 acts in a similar pathway to CDC20 and MAP2, but one that is separate from that of CDPK4^16^. Moreover, it highlights the possibility that *Plasmodium* uses different cell cycle machinery to coordinate various processes during endomitosis, and this will need to be explored in future studies.

## Methods

### Ethics statement

All animal work has passed an ethical review process and was approved by the United Kingdom Home Office. Work was carried out under UK Home Office Project Licenses (40/3344 and 30/3248).

### Generation of transgenic parasites

Gene deletion targeting vectors were constructed using the pBS-DHFR plasmid^11^ and the conditional gene knockdown construct (*P*_*ama1*_*apc3*) was derived from *P*_*ama1*_ (*pSS368*)^28^. *P. berghei* ANKA line 2.34 (for GFP-tagging) or ANKA line 507cl1 (for gene deletion and promoter swap) parasites were transfected by electroporation. Genotypic analysis involved a diagnostic PCR reaction and Southern or Western blot. All of the oligonucleotides used to confirm the mutant parasite lines genetically can be found in Supplementary Table S4.

### Immunoprecipitation and Mass Spectrometry Analysis

Lysates from purified gametocytes of WT-GFP, WT ANKA and APC3-GFP parasite lines activated in ookinete medium for 7 min were incubated with GFP-Trap agarose beads (Chromotek). Bound proteins were digested with trypsin and analysed by LC-MS/MS.

### Two hybrid assay

Budding yeast strain Y2HGold was transformed with the two-hybrid vectors pGADT7 (AD) and pGBKT7 (BD) containing codon-optimised *Plasmodium apc3*, *apc10*, *apc11*, *cdc20* or T-antigen and p53.

### Phenotypic Analysis

Phenotypic analysis was performed at different stages of the parasite life cycle as previously described^2^. Briefly, asexual blood stages and gametocytes were analysed using infected blood smears. Gametocyte activation, zygote formation and ookinete conversion rates were analysed using *in vitro* cultures. For mosquito transmission, triplicate sets of 20 to 60 *Anopheles stephensi* were used. Briefly, exflagellation was examined on day 4 to 5 post-infection. Gametocyte-infected blood was obtained from the tail with a heparinised pipette tip and mixed immediately with 40 µl of ookinete culture medium (RPMI1640 containing 25 mM HEPES, 20% fetal bovine serum, 10 mM sodium bicarbonate, 50 µM xanthurenic acid at pH 7.6). Microgametogenesis was monitored at two different points during mitotic division (8 and 15 mpa). Gametocytes were purified and activated in ookinete medium then fixed and processed for immunofluorescence assay (IFA) with antibodies to a range of different markers. Parasites were visualised on a Zeiss AxioImager M2 microscope fitted with an AxioCam ICc1 digital camera (Carl Zeiss, Inc).

### Deconvolution microscopy

High resolution live cell imaging was performed using an Olympus-based Delta Vision Elite work station fitted with a 100× objective (numerical NA 1.4, oil). Post-acquisition analysis was carried out using Applied Precision software. Images presented are 2D projections of deconvoluted Z-stacks of 0.3 μm optical sections.

### Nuclear DNA content analysis

The nuclear DNA content of gametocytes was analysed by FACS as described previously^16^, using Hoechst dye-fluorescence intensity measured in a LSR-II flow cytometer (Becton Dickinson).

### Quantitative RT-PCR

qRT-PCR reactions used SYBR green fast master mix (Applied Biosystems) and were analysed using an Applied Biosystems 7500 fast machine. Experiments used *hsp70* and *arginine-tRNA synthetase* as reference genes (Supplementary Table S4).

### Electron microscopy

Activated gametocytes at 8, 15 and 30 mpa were fixed in 4% glutaraldehyde in 0.1 M phosphate buffer^16^. Briefly, samples were post fixed in osmium tetroxide, treated *en bloc* with uranyl acetate, dehydrated and embedded in Spurr’s epoxy resin. Thin sections were stained with uranyl acetate and lead citrate prior to examination in a JEOL1200EX electron microscope (Jeol UK Ltd).

## Electronic supplementary material

Supplementary information
