# Author Correction: *Plasmodium* APC3 mediates chromosome condensation and cytokinesis during atypical mitosis in male gametogenesis

**DOI:** 10.1038/s41598-018-30924-6

**Published:** 2018-08-21

**Authors:** Richard J. Wall, David J. P. Ferguson, Aline Freville, Blandine Franke-Fayard, Declan Brady, Mohammad Zeeshan, Andrew R. Bottrill, Sally Wheatley, Andrew M. Fry, Chris J. Janse, Hiroyuki Yamano, Anthony A. Holder, David S. Guttery, Rita Tewari

**Affiliations:** 1 School of Life Sciences, Queens Medical Centre, University of Nottingham, Nottingham, UK; 2 Nuffield Department of Clinical Laboratory Science, University of Oxford, John Radcliffe Hospital, Oxford, UK; 3 Leiden Malaria Research Group, Parasitology, Center of Infectious Diseases, Leiden University Medical Center (LUMC), Leiden, The Netherlands; 4 Protein and Nucleic Acid Chemistry Laboratory, Centre for Core Biotechnology Services, University of Leicester, Leicester, UK; 5 Department of Molecular and Cell Biology, University of Leicester, Leicester, UK; 6 UCL Cancer Institute, University College London, London, UK; 7 The Francis Crick Institute, London, UK; 8 The Leicester Cancer Research Centre, College of Life Sciences, University of Leicester, Leicester, UK; 9 Present Address: The Wellcome Trust Centre for Anti-Infectives Research, School of Life Sciences, University of Dundee, Dundee, UK

Correction to: *Scientific Reports* 10.1038/s41598-018-23871-9, published online 04 April 2018

The Acknowledgements section in this Article contains errors.

“This project was funded by MRC Investigator Award and MRC project grants to RT [G0900109, G0900278, MR/K011782/1]. Work in the HY laboratory was funded by the MRC (MR/M010899/1) and Wellcome Trust (205150/Z/16/Z). AAH was supported by the Francis Crick Institute which receives its core funding from Cancer Research UK (FC001097), the UK Medical Research Council (FC001097), and the Wellcome Trust (FC001097). AMF acknowledges support for his lab from Worldwide Cancer Research (16-0119). We would like to thank Dr Oliver Billker (Sanger Institute) for the P_ama1_/pSS368 construct. We would also like to thank all the members of the RT lab for their technical assistance.”

should read:

“This work was supported by the Medical Research Council [grant numbers G0900109, G0900278, MR/K011782/1, MR/M010899/1] a MRC Investigator Award, MRC project grants to RT and for work in the HY laboratory; and the Wellcome Trust [grant number 205150/Z/16/Z] for work in the HY laboratory. AAH was supported by the Francis Crick Institute which receives its core funding from Cancer Research UK (FC001097), the UK Medical Research Council (FC001097), and the Wellcome Trust (FC001097). AMF acknowledges support for his lab from Worldwide Cancer Research (16-0119). We would like to thank Dr Oliver Billker (Sanger Institute) for the P_ama1_/pSS368 construct. We would also like to thank all the members of the RT lab for their technical assistance.”

