# Supplementary material for: Plasmodium APC3 mediates chromosome condensation and cytokinesis during atypical mitosis in male gametogenesis
Source: Sci Rep. 2018 Apr 4;8:5610. doi: 10.1038/s41598-018-23871-9 (PMC5884774; doi:10.1038/s41598-018-23871-9)
Supplement: Supplementary file 1 — Supplementary information [file 41598_2018_23871_MOESM1_ESM.pdf]

***Plasmodium* APC3 mediates chromosome condensation and cytokinesis during atypical mitosis in male gametogenesis**

Richard J. Wall[1,a], David J. P. Ferguson[2], Aline Freville[1], Blandine Franke-Fayard[3], Declan Brady[1], Mohammad Zeeshan[1], Andrew R. Bottrill[4], Sally Wheatley[1], Andrew M. Fry[5], Chris J. Janse[3], Hiroyuki Yamano[6], Anthony A. Holder[7], David S. Guttery[8] and Rita Tewari[1,*]

Supplementary Figures

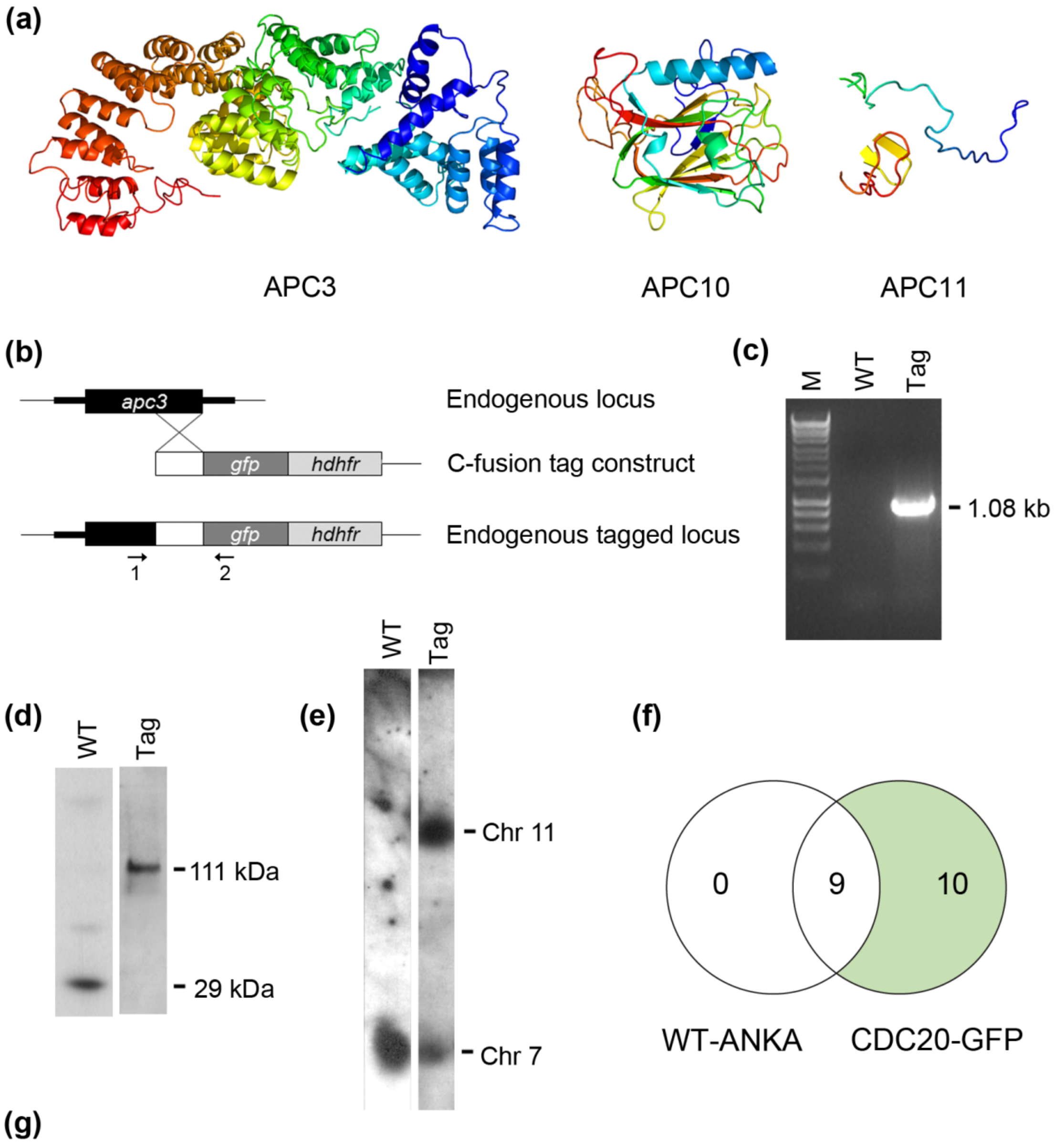

(g)

Accession number	Description
PBANKA_1125600	pyruvate kinase, putative
PBANKA_1459300	actin I
PBANKA_0510600	cell division cycle protein 20 homolog (CDC20)
PBANKA_0805700	heat shock protein 90, putative (HSP90)
PBANKA_1126200	protein DJ-1, putative (DJ1)
PBANKA_1331900	eukaryotic initiation factor 4a, putative (eIF4A)
PBANKA_0941900	histone H4, putative
PBANKA_1423300	DNA/RNA-binding protein Alba 1, putative (ALBA1)
PBANKA_1329300	40S ribosomal protein S3, putative
PBANKA_1217700	ATP-dependent RNA helicase DDX6 (DOZI)

Supplementary Fig. S1. Structural analysis of APC proteins, generation of APC3-GFP parasites and identification of proteins immunoprecipitated with CDC20-GFP.

(a) Predicted structures of *pb*APC3, *pb*APC10 and *pb*APC11 based on Phyre2 folding prediction. APC3, APC10 and APC11 showed identity with human APC3[1] (17% identity), *Saccharomyces cerevisiae* APC10[2] (23% identity) and human APC11[3] (41% identity), respectively. The % identity refers to the likely accuracy of the model.

(b) Schematic representation of the endogenous *apc3* locus, the GFP tagging construct and the recombined apc3 locus following homologous recombination. Arrows 1 and 2 indicate the location of PCR primers used to diagnose integration.

(c) Diagnostic PCR of *apc3-gfp* with primers 1 (IntT114) and 2 (ol492) shows integration of the GFP tag (Tag) and resistance cassette immediately downstream of the *apc3* gene, omitting the endogenous stop codon. Successful integration produced a band of 1.08 kb.

(d) Western blot of APC3-GFP (Tag - 111 kDa) in activated gametocytes compared with WT-GFP (WT - 29 kDa).

(e) Pulse field gel electrophoresis (PFGE) using a *pbdhfr* 3'UTR probe[4]. The probe recognises the endogenous *dhfr* locus on chromosome 7 (WT) and the recombined *apc3* locus on chromosome 11 (Tag) of *P. berghei* ANKA.

(f, g) Analysis of CDC20-GFP by mass spectrometry following immunoprecipitation from a parasite lysate using gametocytes activated for 7 min. Venn diagrams display mean totals of shared and unique proteins compared with WT-GFP and WT-ANKA lines (labelled on graph as WT-ANKA). The table lists unique proteins found only in CDC20-GFP samples. Full results can be found in Supplementary Table S2.

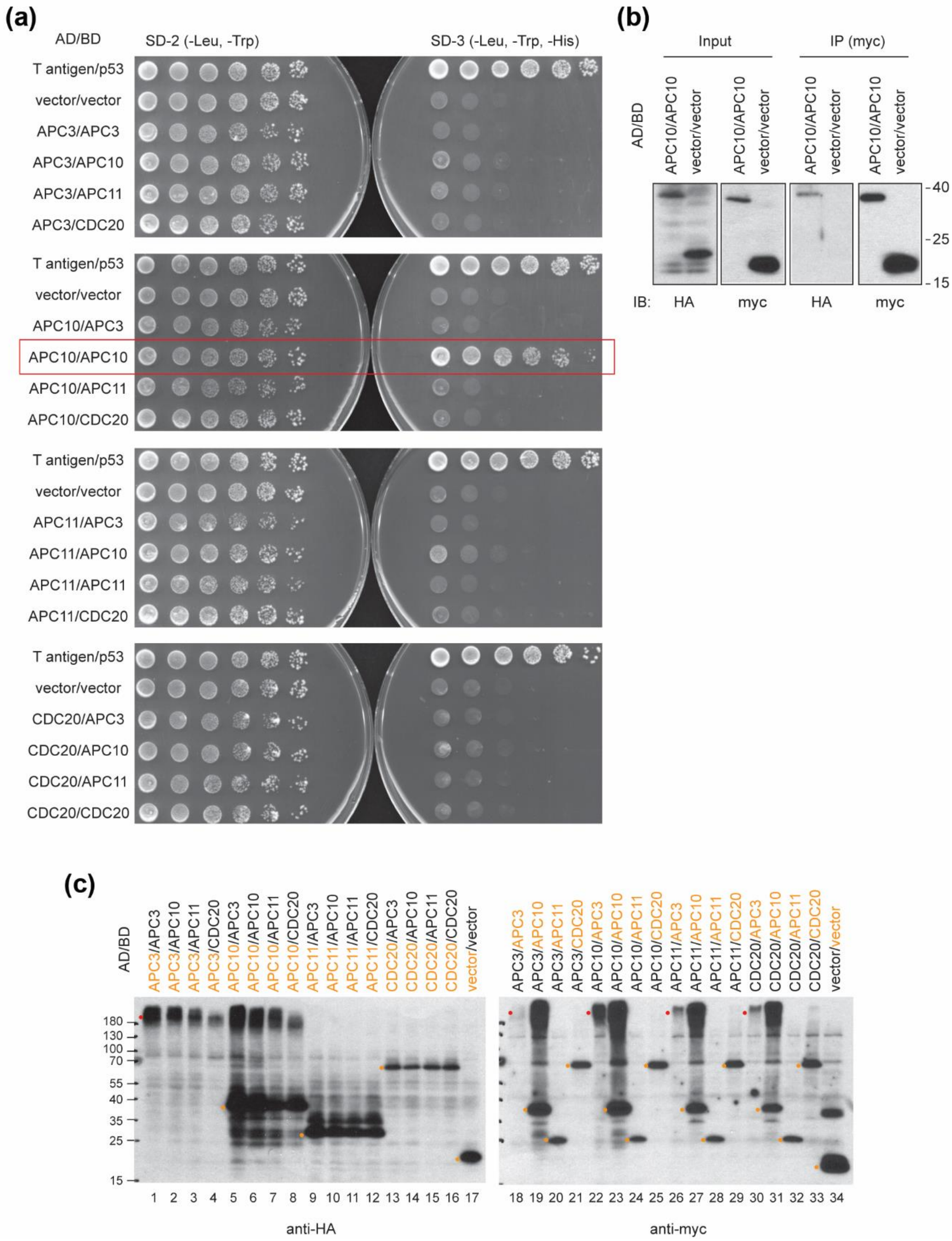
(a)
AD/BD
SD-2 (-Leu, -Trp)
SD-3 (-Leu, -Trp, -His)
T antigen/p53
vector/vector
APC3/APC3
APC3/APC10
APC3/APC11
APC3/CDC20
T antigen/p53
vector/vector
APC10/APC3
APC10/APC10
APC10/APC11
APC10/CDC20
T antigen/p53
vector/vector
APC11/APC3
APC11/APC10
APC11/APC11
APC11/CDC20
T antigen/p53
vector/vector
CDC20/APC3
CDC20/APC10
CDC20/APC11
CDC20/CDC20
(b)
Input
IP (myc)
AD/BD
APC10/APC10
vector/vector
APC10/APC10
vector/vector
APC10/APC10
vector/vector
APC10/APC10
vector/vector
-40
-25
-15
IB: HA myc HA myc
(c)
AD/BD
APC3/APC3
APC3/APC10
APC3/APC11
APC3/CDC20
APC10/APC3
APC10/APC10
APC10/APC11
APC10/CDC20
APC11/APC3
APC11/APC10
APC11/APC11
APC11/CDC20
CDC20/APC3
CDC20/APC10
CDC20/APC11
CDC20/CDC20
vector/vector
APC3/APC3
APC3/APC10
APC3/APC11
APC3/CDC20
APC10/APC3
APC10/APC10
APC10/APC11
APC10/CDC20
APC11/APC3
APC11/APC10
APC11/APC11
APC11/CDC20
CDC20/APC3
CDC20/APC10
CDC20/APC11
CDC20/CDC20
vector/vector
180
130
100
70
55
40
35
25
15
1 2 3 4 5 6 7 8 9 10 11 12 13 14 15 16 17
18 19 20 21 22 23 24 25 26 27 28 29 30 31 32 33 34
anti-HA
anti-myc

Supplementary Fig. S2. Yeast two hybrid assay.

(a) Yeast two hybrid assay. Budding yeast transformants expressing the indicated gene products were spotted in serial dilutions on minimal medium SD-2 (-Leu, -Trp) or SD-3 (-Leu, -Trp, -His). As a positive control, plasmids expressing T antigen and p53 were used.

(b) Pb APC10 forms a dimer. Using a Y2H strain expressing both HA-tagged APC10 and myc-tagged APC10, APC10 was immunoprecipitated with either anti-myc or anti-HA antibody and then bound proteins were detected with anti-HA or anti-myc antibody, respectively.

(c) Cell extracts from Y2H strains used were immunoblotted with anti-HA or anti-myc antibody. Plasmid pGADT7 expresses Gal4 activation domain (AD) with a HA tag whereas plasmid pGBKT7 expresses Gal4 DNA binding domain (BD) with a myc tag. AD- or BD-APC10, APC11 and CDC20 bands in blots are highlighted with an orange dot. Neither AD- nor BD-APC3 was clearly detected at the expected molecular weight (MW) although some signals were observed at higher MW area (marked with red dots). APC3 may be aggregated or poorly expressed even using a 'yeast' codon-optimized gene.

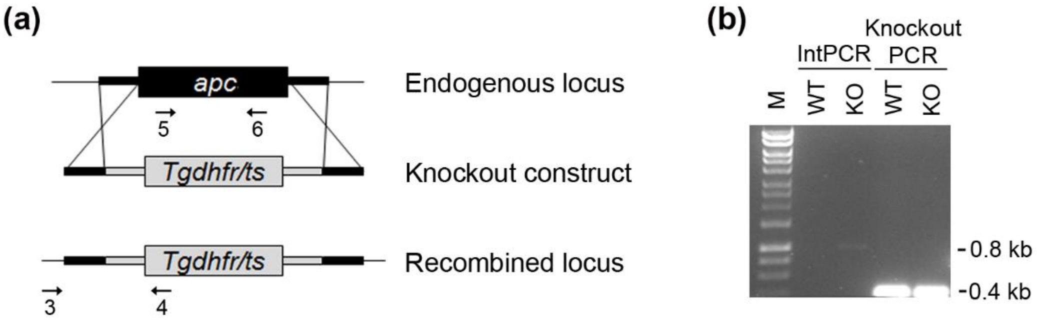

Supplementary Fig. S3. Attempted gene deletion.

(a) Schematic representation of the endogenous *apc3* locus, the targeting knockout construct and the recombined *apc3* locus following successful double homologous cross-over recombination. Arrows 3 and 4 indicate PCR primers used to confirm successful integration in the *apc3* locus and arrows 5 and 6 indicate PCR primers used to show deletion of the *apc3* gene following recombination.

(b) Diagnostic PCR of an *apc3* gene deletion attempt. Integration of the targeting construct and amplification with primers 3 (IntN73) and 4 (ol248) would be revealed by a band of 0.8 kb in attempted KO but not in WT (lanes 2 and 3 – no band present in either lane), whilst the presence of the *apc3* gene and amplification using primers 5 (N73KO1) and 6 (N73KO2) gives a band of 0.4kb in both samples (lanes 4 and 5). It was not possible to generate gene deletion mutants for any of the APC/C subunits.

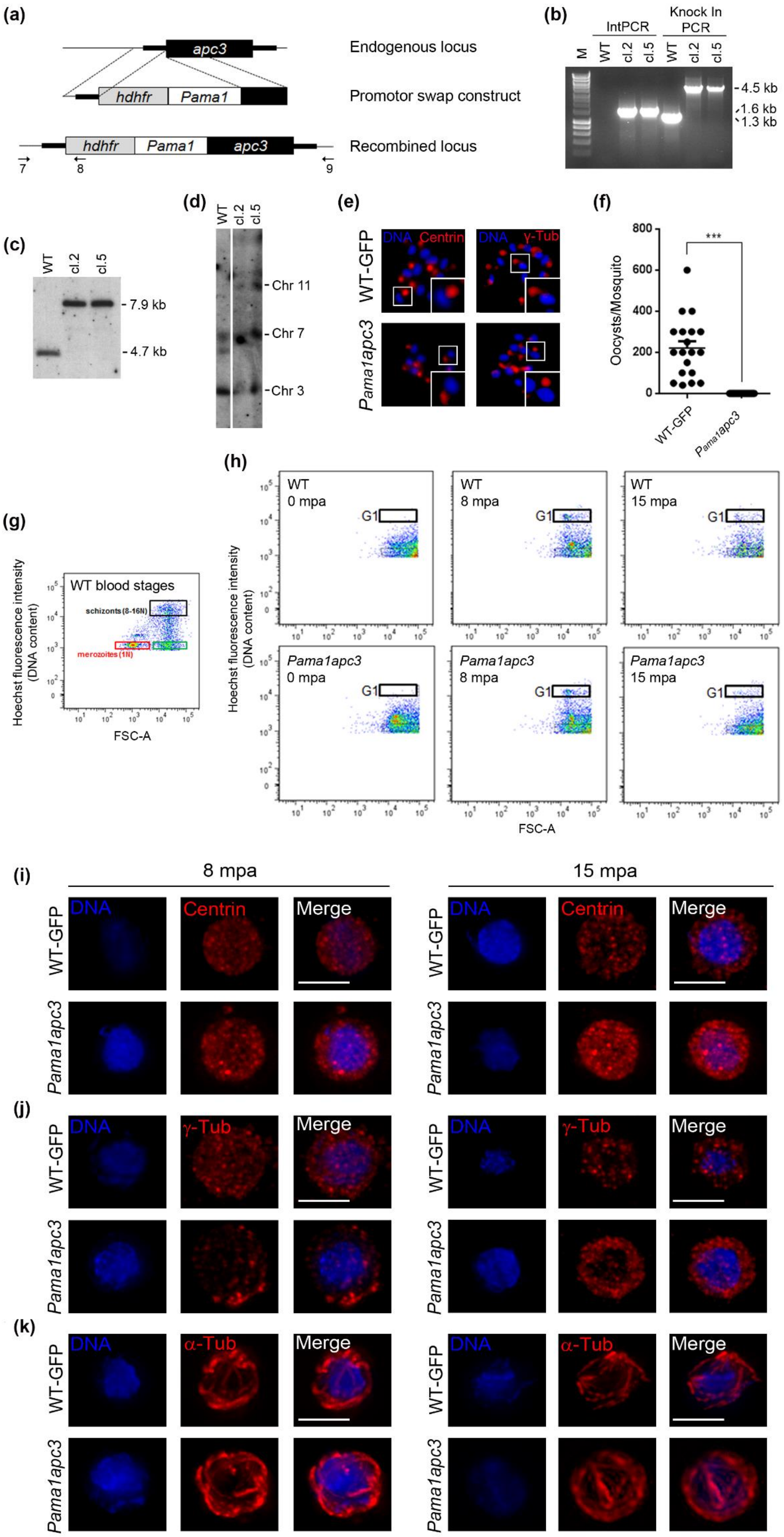
(a)
apc3
Endogenous locus
hdhfr
Pama1
Promotor swap construct
hdhfr
Pama1
apc3
Recombined locus
7
8
9
(b)
IntPCR
Knock In PCR
M
WT
cl.2
cl.5
WT
cl.2
cl.5
4.5 kb
1.6 kb
1.3 kb
(c)
WT
cl.2
cl.5
7.9 kb
4.7 kb
(d)
WT
cl.2
cl.5
Chr 11
Chr 7
Chr 3
(e)
WT-GFP
Pama1apc3
DNA Centrin
DNA γ-Tub
(f)
Oocysts/Mosquito
***
WT-GFP
Pama1apc3
(g)
WT blood stages
schizonts (8-16N)
merozoites (1N)
Hoechst fluorescence intensity (DNA content)
FSC-A
(h)
WT 0 mpa
WT 8 mpa
WT 15 mpa
Pama1apc3 0 mpa
Pama1apc3 8 mpa
Pama1apc3 15 mpa
G1
Hoechst fluorescence intensity (DNA content)
FSC-A
(i)
8 mpa
15 mpa
WT-GFP
Pama1apc3
DNA
Centrin
Merge
(j)
DNA
γ-Tub
Merge
(k)
DNA
α-Tub
Merge

Supplementary Fig. S4. Generation and functional analysis of *P*$_{ama1}$*apc3* parasites.

(a) Schematic of the endogenous *apc3* locus and the promoter swap construct. Arrows 7 and 8 indicate PCR primers used to show integration of the targeting construct and Arrow 9 (in combination with Arrow 7) indicates primers used to show successful knock-in of the construct.

(b) Diagnostic PCR to show integration of the promotor swap construct into the *apc3* locus. Primer 7 (5'IntPTD003) with primer 8 (5'IntPTD) were used to determine successful integration of the selectable marker resulting in a band of 1.6kb (lanes 2 to 4). Primer 7 and primer 9 (3'IntPTD003) were used to show complete knock-in of the construct with a band at 4.5kb and the absence of a band at 1.3kb (endogenous gene) resulting in complete knock-in of the construct in two clones, 2 and 5 (lanes 5 to 7).

(c) Southern blot analysis of WT and *P*$_{ama1}$*apc3* parasite (cl.2 and cl.5) genomic DNA following *Pac*I digestion. A probe specific for the *apc3* 3'UTR bound to a 4.7 kb band in WT and to a 7.9 kb band in *P*$_{ama1}$*apc3* parasite DNA.

(d) Pulse Field Gel Electrophoresis (PFGE) using a *pbdhfr* 3'UTR probe. The probe recognises the endogenous *dhfr* locus on chromosome 7, the *gfp* cassette integrated in the 230p locus of the GFP-transgenic parasites used for transfection (WT) (chromosome 3) and the recombined *apc3* locus on chromosome 11 (cl.2 and cl.5).

(e) IFA localisation of either centrin or gamma-tubulin of *P*$_{ama1}$*apc3* or WT-GFP schizonts compared with DNA staining.

(f) Number of oocysts per mosquito after 14 days maturation for *P*$_{ama1}$*apc3* and WT-GFP parasites. n=20 from two independent experiments.

(g and h) Determination of DNA content of asexual blood stages, and purified male gametocytes at 0, 8 and 15 mpa by FACS analysis of Hoechst-stained parasites. Gate G1 contains activated male gametocytes with an 8N DNA content. The number of *P*$_{ama1}$*apc3*

activated male gametocytes with an 8N DNA content is comparable to that of WT-GFP parasites.

(i) centrin, (j) γ-tubulin and (k) α-tubulin localisation with specific antibodies in P_{ama1}*apc3* or WT-GFP microgametocytes activated for 8 or 15 min. DAPI DNA staining and the merged images are also shown. Scale bar = 5 µm.

__Supplementary Tables__

Supplementary Table S1. List of main protein hits in the APC3-GFP immunoprecipitation experiments.

Gene Products	Gene ID	Weight	Experiment 1		Experiment 2		Experiment 3		Experiment 4	
			GFP	APC3	GFP	APC3	ANKA	APC3	ANKA	APC3
heat shock protein, putative (HSP70)	PBANKA_0711900	75 kDa	15	26	7	31	3	31	3	10
tubulin beta chain, putative	PBANKA_1206900	50 kDa	15	9	11	18	11	25	14	19
enolase, putative (ENO)	PBANKA_1214300	49 kDa	2	8	3	11	9	26	10	11
**anaphase-promoting complex 3**	PBANKA_1101500	82 kDa		18		27		17		9
pyruvate kinase, putative	PBANKA_1125600	56 kDa		11	6	19		23		11
elongation factor 1 alpha (EF-1alpha)	PBANKA_1133300	49 kDa	4	8	2	13	7	17	7	9
Cluster of alpha tubulin 2	PBANKA_0522700	50 kDa	3	3	6	14	5	19	3	11
heat shock protein 90	PBANKA_0805700	83 kDa		13		18		18		3
ribonucleoside-diphosphate reductase	PBANKA_0611600	97 kDa	2				4	26	5	14
glyceraldehyde dehydrogenase	PBANKA_1326400	37 kDa				6	4	24	6	9
actin I	PBANKA_1459300	42 kDa	4		2	15	5	16		6
alpha tubulin 2	PBANKA_0522700	50 kDa	2	2	3	5	5	15	3	11
fructose-bisphosphate aldolase 2	PBANKA_1308600	40 kDa			4	4	4	22	4	4
heat shock protein 70, putative	PBANKA_0818900	72 kDa		8		11		12		
ubiquitin-like protein, putative	PBANKA_0823000	161 kDa		10		17				
alpha tubulin 1	PBANKA_0417700	50 kDa				1		14		7
ATP-dependent RNA Helicase (DOZI)	PBANKA_1217700	49 kDa	2			2		13		3
high molecular weight rhoptry protein 2	PBANKA_0830200	160 kDa		11		9				
14-3-3 protein (14-3-3I)	PBANKA_0712600	30 kDa					3	10		5
eukaryotic initiation factor 4a, putative	PBANKA_1331900	45 kDa				5		8		4
60S ribosomal protein L4, putative	PBANKA_1106700	46 kDa				11				5
protein disulfide isomerase	PBANKA_0702800	55 kDa				4		12		
40S ribosomal protein S2B, putative	PBANKA_0510900	30 kDa				4	3	8		

elongation factor 2, putative	PBANKA_1314800	93 kDa		12		3				
macrophage migration inhibitory factor	PBANKA_1444000	13 kDa					3	4	4	4
HSP40, subfamily A, putative	PBANKA_0610900	48 kDa				13				
DNA/RNA-binding protein Alba 4	PBANKA_1360300	42 kDa		2		6		5		
karyopherin beta, putative	PBANKA_1238800	128 kDa		4		8				
Plasmodium exported protein	PBANKA_1229000	126 kDa		6		5				
hexokinase, putative	PBANKA_1122900	55 kDa		2		9				
adenosylhomocysteinase	PBANKA_1235600	54 kDa				3		6		
plasmepsin IV (PM4)	PBANKA_1034400	50 kDa				4		4		
polyubiquitin, putative	PBANKA_0610300	26 kDa		4		2				
hypoxanthine-guanine transferase	PBANKA_1210800	27 kDa						6		
RhopH3, putative	PBANKA_0416000	105 kDa		3		2				
leucine aminopeptidase, putative	PBANKA_1309900	72 kDa		3		2				
conserved Plasmodium protein	PBANKA_1214700	112 kDa						5		
NIMA related kinase 2 (NEK2)	PBANKA_1240700	33 kDa			2	2				
osmiophilic body protein (G377)	PBANKA_1463000	309 kDa				4				
trailer hitch homolog, putative (CITH)	PBANKA_1301300	39 kDa								3
conserved Plasmodium protein	PBANKA_1137800	291 kDa				3				
cytoadherence linked asexual protein	PBANKA_1400600	152 kDa				3				
Hsc70-interacting protein (HIP)	PBANKA_1242300	48 kDa				3				
60S ribosomal protein	PBANKA_0918000	35 kDa				3				
40S ribosomal protein S11	PBANKA_0807600	19 kDa						3		
TCP-1/cpn60 chaperonin family	PBANKA_0916200	60 kDa				2				
polyadenylate-binding protein	PBANKA_1439200	93 kDa				2				
chaperone, putative	PBANKA_0107300	61 kDa				2				
T-complex protein beta subunit	PBANKA_0405200	59 kDa				2				

Weight: Molecular weight; GFP: WT-GFP; ANKA: WT-ANKA; APC3: APC3-GFP. Proteins are ordered based on the number of unique peptides.

Supplementary Table S2. List of main protein hits in the CDC20-GFP immunoprecipitation experiments.

Gene Product	Gene ID	Weight	Experiment 1		Experiment 2	
			ANKA	CDC20	ANKA	CDC20
tubulin beta chain, putative	PBANKA_1206900	50 kDa	11	20	14	18
ribonucleoside-diphosphate reductase	PBANKA_0611600	97 kDa	4	17	5	14
enolase, putative (ENO)	PBANKA_1214300	49 kDa	9	16	10	4
fructose-bisphosphate aldolase 2 (ALDO2)	PBANKA_1308600	40 kDa	4	10	4	12
glyceraldehyde-3-phosphate dehydrogenase	PBANKA_1326400	37 kDa	4	10	6	7
elongation factor 1-alpha (EF-1alpha)	PBANKA_1133300	49 kDa	7	10	7	3
heat shock protein 70 (HSP70)	PBANKA_0711900	75 kDa	3	11	3	9
alpha tubulin 2	PBANKA_0522700	50 kDa	5	9	3	7
pyruvate kinase, putative	PBANKA_1125600	56 kDa		10		11
Cluster of actin I	PBANKA_1459300	42 kDa	5	7		7
actin I	PBANKA_1459300	42 kDa		7		7
40S ribosomal protein S2, putative (RPS2)	PBANKA_0510900	30 kDa	3	7		4
14-3-3 protein (14-3-3I)	PBANKA_0712600	30 kDa	3	5		5
macrophage migration inhibitory factor (MIF)	PBANKA_1444000	13 kDa	3	3	4	2
**cell division cycle protein 20 homolog (CDC20)**	PBANKA_0510600	63 kDa		3		8
heat shock protein 90, putative (HSP90)	PBANKA_0805700	83 kDa		6		4
protein DJ-1, putative (DJ1)	PBANKA_1126200	21 kDa				5
eukaryotic initiation factor 4a, putative (eIF4A)	PBANKA_1331900	45 kDa		2		3
histone H4, putative	PBANKA_0941900	11 kDa		2		3
DNA/RNA-binding protein Alba 1, putative (ALBA1)	PBANKA_1423300	27 kDa		2		2
40S ribosomal protein S3, putative	PBANKA_1329300	25 kDa		2		2
ATP-dependent RNA helicase DDX6 (DOZI)	PBANKA_1217700	49 kDa				3
40S ribosomal protein S18, putative	PBANKA_0922100	18 kDa		2		
hypoxanthine-guanine phosphoribosyltransferase	PBANKA_1210800	27 kDa		2		
60S ribosomal protein L4, putative (RPL4)	PBANKA_1106700	46 kDa		2		
Cluster of histone H2A variant, putative (H2A.Z)	PBANKA_1217600	16 kDa				2

histone H2A variant, putative (H2A.Z)	PBANKA_1217600	16 kDa				2
thioredoxin peroxidase 1 (TPx1)	PBANKA_1302800	22 kDa				2
protein disulfide isomerase related protein, putative	PBANKA_0914300	49 kDa		2		
male development gene 1 (MDV1)	PBANKA_1432200	24 kDa		2		
40S ribosomal protein S20e, putative	PBANKA_1201900	14 kDa		2		
40S ribosomal protein S11, putative	PBANKA_1231000	16 kDa				2
40S ribosomal protein S29, putative	PBANKA_0803400	6 kDa		2		
elongation factor 2, putative (eEF2)	PBANKA_1314800	93 kDa		2		
chromatin assembly factor 1 protein WD40 domain	PBANKA_0203000	51 kDa				
60S ribosomal protein L10, putative	PBANKA_1028400	25 kDa				

Weight: Molecular weight; GFP: WT-GFP; ANKA: WT-ANKA; CDC20: CDC20-GFP. Proteins are ordered based on the number of unique peptides.

Supplementary Table S3: Quantification of the immune-localisation studies with APC3-GFP activated gametocytes (15 min).

Localisation	Count	Percentage
**1 dot**	37	52
**2 dots**	12	17
**> 2 dots**	5	7
**Inconclusive**	10	14
**None**	7	10

Supplementary Table S4. Primers used in this study (5' – 3').

Name	Sequence	Notes
T1141	CCCCGGTACCCAACTTGTTATTGGCACAAAAAGG	KpnI site underlined
T1142	CCCCGGGCCCTTCTAATAACGAGTTCTCAAAATCATATATATC	ApaI site underlined
IntT114	CGAAGTTATTCAAGATATTGACTG	
ol492	ACGCTGAACTTGTGGCCG	
N0731	CCCCGGGCCCCTCGATGTGATATATGTGTAGAC	ApaI site underlined
N0732	GGGGAAGCTTCATTTAAATCATCATAGTTTAAATAAGGCTC	HindIII site underlined
N0733	CCCCGAATTCGATCTCATAAGAGCATTTATTCGTTG	EcoRI site underlined
N0734	GGGGTCTAGAGTATAACTGAATAACTATGCATTTCATTAG	XbaI site underlined
N0781	CCCCGGGCCCGCATACTCGAATATCATATATG	ApaI site underlined
N0782	GGGGAAGCTTCTGCTTGATAATTTCCAAATTC	HindIII site underlined
N0783	CCCCGAATTCGGTCCTAATTATTCTGTTTACAAATAC	EcoRI site underlined
N0784	GGGGTCTAGAGATAATTTTAAAGAAGAATTTGCCAGTG	XbaI site underlined
N0771	CCCCGGGCCCGATGCGAAACATTGACGTTTCAGC	ApaI site underlined
N0772	GGGGAAGCTTCATTTCCACCTTGCAACTGCATG	HindIII site underlined
N0773	CCCCGAATTCGTTAACGTGCCCATGTTGTCGAGC	EcoRI site underlined
N0774	GGGGTCTAGAGGGGGAAGTAGGTTTTATAAGATGG	XbaI site underlined
IntN73	CCTCTTAAAGGATCTTGATTATTTTG	
IntN78	GCATAAATGGAAATATTCGCTTGC	
IntN77	CCGCAGTTATATTTACATTACTTCATAG	
ol248	GATGTGTTATGTGATTAATTCATACAC	
N73KO1	TCTAAAACGGGCATCAACGA	
N73KO2	AGTTCGCAGCAATTTCTTTCTT	
PTD0031	GAGACCGCGGCCTCTTAAAGGATCTTGATTATTTTGTA	SacII site underlined

PTD0032	GAGACTGCAGTCTCAAATCGAAAAATAAAATATAATAAAATGAG	PstI site underlined
PTD0033	GAGACTCGAGATGAAGAAACAGAATAACAAGATGCC	XhoI site underlined
PTD0034	GAGAGCGGCCGCGATATCAAACCATTCTTCCTTTGCTTCTT	NotI site underlined
5’IntPTD003	GTATTCATATTAATATTACACACATGCG	
3’IntPTD003	CCATGTAACATTAATAACTTTCGAAATG	
5'IntPTD	TCTACTTTATTTGCTAATTCTGG	
3’IntPTDama1	TGTGTATATATAAGTATTGTATGGTAATTG	
hsp70 FW	GTATTATTAATGAACCCACCGCT	PBANKA_0818900
hsp70 RV	GAAACATCAAATGTACCACCTCC	
arginyl FW	TTGATTCATGTTGGATTTGGCT	PBANKA_1434200
arginyl RV	ATCCTTCTTTGCCCTTTCAG	
apc3 FW	TGAAAGTTCACAGGATGTCAACAA	PBANKA_1101500
apc3 RV	AACACACAATCCGAAGGTGCA	
apc10 FW	ATACATACTGGCAATCATCGTC	*PBANKA_1433200*
apc10 RV	GGGTATATGATTCGTCAAGCA	
apc11 FW	CACAATTTGTATAAGACCGGGA	*PBANKA_1123400*
apc11 RV	TTGTAATACCAATCTGCTCGA	
map2 FW	AATGAAGAACCAGGGCCA	PBANKA_0933700
map2 RV	ACCATCTAGTAACTACATGGCT	
cdpk4 FW	AAATGTTGATGTACACAAGTGC	PBANKA_0615200
cdpk4 RV	ATGTTCTAATGCATCTCTTGCT	
cdc20 FW	ATGTTTGGTAACTATTTGGCGG	PBANKA_0510600
cdc20 RV	ATCCCATATTTCTACTGCACCA	

Supplementary procedures

Protein domain analysis

Protein domains were identified for *pb*CDC20 previously[5]. ScanProsite was used to identify the domains of the other APC/C components[6] and a TPR domain predictor (TPRpred) was used for sequence analysis of *pb*APC3[7], Protein domain figures were generated using MyDomains software[8]. Homology modelling was performed using Phyre2 software[9].

Generation of transgenic parasites

All of the oligonucleotides used to genetically confirm the mutant parasite lines can be found in Table S3. For GFP-tagging of *apc3* by single homologous recombination, a region of *apc3* (PBANKA_1101500) downstream of the ATG start codon, amplified using primers T1141 and T1142, was used to generate the construct as described previously[5]. The gene deletion targeting vectors for Δ*apc3*, Δ*apc10* (PBANKA_1433200) *and* Δ*apc11* (PBANKA_1123400) were constructed using the pBS-DHFR plasmid, which contains polylinker sites flanking a *T. gondii dhfr/ts* expression cassette conveying resistance to pyrimethamine, as described previously[4]. The three constructs were transfected in at least 4 independent experiments (a positive control of the APC3-GFP construct was also used). The conditional knockdown construct (P_{ama1}*apc3*) was derived from P_{ama1} (*pSS368*) where APC3 was placed under the control of *ama1* promoter as described previously[10]. PCR primers PTD0031 and PTD0032 were used to generate a 609 bp fragment of 5′ sequence upstream of *apc3, directly before the start codon*. A 531 bp fragment generated with primers PTD0033 and PTD0034 from the 3′ flanking region of *apc3* was then inserted downstream of the *ama1* promoter region. *P. berghei* ANKA line 2.34 (for GFP-tagging) or ANKA line 507cl1 (for gene deletion and promoter trap) parasites were transfected by electroporation. Six-to-eight week old female Tuck-Ordinary (TO) (Harlan) outbred mice were used for all experiments.

Genotypic analysis of mutants

For the C-terminal fusion GFP tagged parasites, diagnostic PCR confirmed correct integration and Western blot confirmed GFP expression and the correct protein size. For the

gene deletion and promoter swap parasites, a diagnostic PCR reaction and Southern blot were used. These approaches are similar to those described previously[10,11].

Yeast two hybrid assay

Budding yeast strain Y2HGold was transformed with the 2-hybrid vectors pGADT7 (AD) and pGBKT7 (BD) containing codon optimised *Plasmodium apc3*, *apc10*, *apc11*, *cdc20* or T-antigen and p53. As each vector contains a nutritional marker, LEU2 and TRP1, respectively, transformants were selected on SD medium lacking leucine and tryptophan (SD-2: -Leu, -Trp). In order to examine protein interactions, the transformants were spotted out on SD medium lacking leucine, tryptophan and histidine (SD-3: -Leu, -Trp, -His) in serial dilutions and incubated at 30˚C for 3 to 4 days. Each plate contained a positive control (T antigen/p53) and a negative control (pGADT7 vector/pGBKT7 vector).

Purification of schizonts, gametocytes and ookinetes

Purification of gametocytes was achieved using a modified version of a previous protocol [12]. Mice were treated by intraperitoneal injection of 0.1 ml of phenylhydrazine (6 mg/ml) (Sigma) in PBS to encourage reticulocyte formation four days prior to infection with parasites. Four days after parasites injection, recipient mice were treated with sulfadiazine (Sigma) at 20 mg/L in their drinking water for two days to eliminate asexual blood stage parasites. On day six post-injection (p.i), mice were bled by cardiac puncture into heparin and gametocytes separated from uninfected erythrocytes on a 48% NycoDenz gradient (27.6% w/v NycoDenz in 5 mM Tris-HCl, pH 7.20, 3 mM KCl, 0.3 mM EDTA) in coelenterazine loading buffer (CLB), containing PBS, 20 mM HEPES, 20 mM Glucose, 4 mM sodium bicarbonate, 1 mM EGTA, 0.1% w/v bovine serum albumin, pH 7.25. Gametocytes were harvested from the interface and washed twice in RPMI 1640 ready for activation of gamete formation. Blood cells from day 5 p.i mice were placed in culture (40 ml RPMI 1640, 8 ml foetal bovine serum, 0.5 ml penicillin and streptomycin; per 0.5 ml blood) for 24 h at 37°C for schizont development (with rotation at 100 rpm) and at 20°C for ookinete production as described above. Schizonts and

ookinetes were purified on 60% and 63% NycoDenz gradients, respectively and harvested from the interface and washed.

Immunoprecipitation and Mass Spectrometry Analysis

Purified gametocytes of WT-GFP, WT ANKA and APC3-GFP parasite lines were activated in ookinete medium for 7 min and suspended in 200 µl ice-cold lysis buffer (10mM Tris/Cl pH 7.5, 150 mM NaCl, 0.5mm EDTA, 0.5% NP40). The cells were incubated on ice for 30 min with extensive mixing by pipetting every 10 min. The cell lysates were centrifuged at 13000 rpm for 10 min at 4˚ C. The clear lysates were incubated with GFP-Trap agarose beads (Chromotek) for 1h and unbound proteins were removed with wash buffer (10mM Tris/Cl pH 7.5, 150 mM NaCl, 0.5mm EDTA). Beads were re-suspended in 5 volumes of SDS-PAGE sample buffer and boiled for 10 min prior to fractionation of the proteins by SDS-PAGE. Proteins were run 15 mm into a 4-12% SDS-polyacrylamide gel, and then excised using a clean scalpel blade. These samples were digested with trypsin and analysed by LC-MS/MS.

Live imaging and Immunofluorescence assay

Gametocytes were purified and activated in ookinete medium then fixed and processed for IFA with different markers. *P. berghei* schizonts and gametocytes were collected from parasite-infected blood (as described above). For live imaging, parasites were stained with Hoechst 33342 DNA stain before being mounting for fluorescent microscopy. For immuno-localisation, parasites were suspended in 4% paraformaldehyde (PFA) (Sigma) diluted in PBS (schizonts) or microtubule stabilising buffer (MTSB; gametocytes) for 10-15 min and added to poly-L-lysine coated slides. Immunocytochemistry was performed using primary GFP-specific rabbit monoclonal antibody (mAb) (Invitrogen-A1122; used at 1:250), primary anti-centrin mAb (Millpore-04-1624; used at 1:200), primary mouse anti-alpha tubulin mAb (Sigma-T9026; used at 1:500: gametocytes, 1:1000: schizonts), primary anti-gamma tubulin antibody (Sigma-T6557; used at 1:500) for 1 h. Secondary antibodies were Alexa 568 conjugated anti-mouse IgG (Invitrogen-A11004) and Alexa 488 (Invitrogen-A11034) conjugated anti-rabbit IgG (used at 1 in 1000). The slides were then mounted in Vectashield

with DAPI (Vector Labs) for fluorescence microscopy. Parasites were visualised on a Zeiss AxioImager M2 microscope fitted with an AxioCam ICc1 digital camera (Carl Zeiss, Inc).

Nuclear DNA content analysis

The nuclear DNA content was analysed by FACS as described previously[5]. Briefly, purified gametocytes were activated with ookinete medium for the specified time. Activated cells were then pelleted by centrifugation (5 sec; 10,000 rpm), fixed in 0.25% glutaraldehyde/PBS solution and stained with 2 µM Hoechst-33258. The Hoechst-fluorescence intensity (DNA content) was analysed by FACS using a LSR-II flow cytometer (Becton Dickinson) with the following filters (parameters/thresholds): UB 440/40 (Hoechst) (400/5000); FSC (250/2000); SSC (200/5000). A total of 100,000 cells was analysed per sample and all measurements were performed on triplicate samples. To determine the Hoechst-fluorescence intensity (DNA content) from the populations of activated macro- and micro- gametocytes, gates were set as described previously[5]. Data processing and analysis was performed using the program FlowJo (http://www.flowjo.com).

Quantitative RT-PCR

Total RNA was isolated from purified parasites using an RNeasy purification kit (Qiagen). cDNA was synthesised using an RNA-to-cDNA kit (Applied Biosystems) allowing quantification from 150 ng of total RNA. qRT-PCR reactions used SYBR green fast master mix (Applied Biosystems) and were analysed using an Applied Biosystems 7500 fast machine with the following cycling conditions: 95 °C for 20 sec followed by 40 cycles of 95 °C for 3 sec; 60 °C for 30 sec. Gene expression in wild type parasites was determined using the Pfaffl method[13]. Relative quantification in the mutant line was normalised against wild-type expression using the ΔΔCt method. Both experiments used *hsp70* and *arginine-tRNA synthetase* as reference genes. Three biological replicates were used for each stage (each with two technical replicates). The primers used are described in Supplementary Table S3. Statistical analyses were performed using Excel and GraphPad Prism (GraphPad Software). For wild type expression an unpaired Student's t-test was performed between *apc3* NAG

and the other APC/C components. For relative gene expression, a Student's unpaired *t*-test between wild type and mutant expression levels was used.

Supplementary references

1 Yamaguchi, M. *et al.* Structure of an APC3–APC16 Complex: Insights into Assembly of the Anaphase-Promoting Complex/Cyclosome. *Journal of Molecular Biology*.

2 Au, S. W., Leng, X., Harper, J. W. & Barford, D. Implications for the ubiquitination reaction of the anaphase-promoting complex from the crystal structure of the Doc1/Apc10 subunit. *Journal of molecular biology* **316**, 955-968, (2002).

3 Chang, L., Zhang, Z., Yang, J., McLaughlin, S. H. & Barford, D. Atomic structure of the APC/C and its mechanism of protein ubiquitination. *Nature* **522**, 450-454, (2015).

4 Tewari, R. *et al.* The Systematic Functional Analysis of Plasmodium Protein Kinases Identifies Essential Regulators of Mosquito Transmission. *Cell Host & Microbe* **8**, 377-387, (2010).

5 Guttery, D. S. *et al.* A Putative Homologue of CDC20/CDH1 in the Malaria Parasite Is Essential for Male Gamete Development. *PLoS Pathog* **8**, e1002554, (2012).

6 de Castro, E. *et al.* ScanProsite: detection of PROSITE signature matches and ProRule-associated functional and structural residues in proteins. *Nucleic acids research* **34**, W362-W365, (2006).

7 Karpenahalli, M. R., Lupas, A. N. & Soding, J. TPRpred: a tool for prediction of TPR-, PPR- and SEL1-like repeats from protein sequences. *BMC bioinformatics* **8**, 2, (2007).

8 Hulo, N. *et al.* The 20 years of PROSITE. *Nucleic acids research* **36**, D245-249, (2008).

9 Kelley, L. A. & Sternberg, M. J. Protein structure prediction on the Web: a case study using the Phyre server. *Nature protocols* **4**, 363-371, (2009).

10 Sebastian, S. *et al.* A Plasmodium Calcium-Dependent Protein Kinase Controls Zygote Development and Transmission by Translationally Activating Repressed mRNAs. *Cell host & microbe* **12**, 9-19, (2012).

11 Guttery, David S. *et al.* Genome-wide Functional Analysis of Plasmodium Protein Phosphatases Reveals Key Regulators of Parasite Development and Differentiation. *Cell Host & Microbe* **16**, 128-140, (2014).

12 Beetsma, A. L., van de Wiel, T. J., Sauerwein, R. W. & Eling, W. M. Plasmodium berghei ANKA: purification of large numbers of infectious gametocytes. *Experimental parasitology* **88**, 69-72, (1998).

13 Pfaffl, M. W. A new mathematical model for relative quantification in real-time RT-PCR. *Nucleic Acids Res* **29**, e45, (2001).
